# Organic textile waste as a resource for sustainable agriculture in arid and semi-arid areas

**DOI:** 10.1007/s13280-016-0822-5

**Published:** 2016-09-24

**Authors:** Bo G. Eriksson

**Affiliations:** Department of Sociology and Work Science, Retired from University of Gothenburg, Sveagatan 29, 413 14 Gothenburg, Sweden

**Keywords:** Agriculture, Arid and semi-arid, Carbon sequestration, Soil organic matter, Textile, Waste

## Abstract

New vegetation in barren areas offers possibilities for sequestering carbon in the soil. Arid and semi-arid areas (ASAs) are candidates for new vegetation. The possibility of agriculture in ASAs is reviewed, revealing the potential for cultivation by covering the surface with a layer of organic fibres. This layer collects more water from humidity in the air than does the uncovered mineral surface, and creates a humid environment that promotes microbial life. One possibility is to use large amounts of organic fibres for soil enhancement in ASAs. In the context of the European Commission Waste Framework Directive, the possibility of using textile waste from Sweden is explored. The costs for using Swedish textile waste are high, but possible gains are the sale of agricultural products and increased land prices as well as environmental mitigation. The findings suggest that field research on such agriculture in ASAs should start as soon as possible.

## Introduction

In the 1970s, the United Nation’s FAO suggested research on the use of organic waste from the industrial world to improve soil productivity in developing countries (Singh 1975; Flaig et al. 1977). The perspective in this study is to promote field research on the use of organic textile waste for agricultural purposes in arid and semi-arid areas (ASAs). Two main limits to agriculture in ASAs are the shortage of water and the shortage of soil organic matter (SOM). The main form of environmental mitigation would first be the conservation of carbon in the textiles compared with the current burning (Östlund et al. 2015; Zamani et al. 2015); second, the sequestration of soil carbon (SSC) from atmospheric CO_2_ by plant life in previously barren land (Zhang et al. 2009; Poeplau and Don 2015; Paustian et al. 2016); and third, increased water absorption (Jacobs et al. 1999; Agam and Berliner 2004, 2006; Fischer et al. 2012). Sustainable cultivation would also offer livelihoods to human populations. As no research has been conducted on the use of textiles for increasing SOM, the arguments are supported by research on similar practices, governmental reports, reports from NGOs, and examples from trials with less certain evidence. In the literature, total organic carbon (TOC) and soil organic carbon (SOC) are often reported as alternatives to SOM. There is no fixed relation among SOM, TOC, and SOC. This relation depends on the density of carbon in SOM. The arguments proposed in this context could be extended to the worldwide mass of organic fibre waste, but in this study, the focus is on textile waste in Sweden.

The outline of the text starts with possible functions of textile waste for water circulation and soil carbon sequestration in ASAs followed by the use of textile waste from Sweden as a possible resource in these areas. These possibilities are discussed.

## Considerations

### Water

Using ground and surface water to increase plant life in current deserts is problematic. If there is no permanent refilling of the ground water supply, then this resource is limited and thus not sustainable (Mollison 1988: 309; Olsson 2015: 114–117). The risk with surface water, such as irrigation with river water, is that the soil becomes salinized and infertile (Olsson 2015: 96). All water management requires energy (Olsson 2015), and it is more desirable if the transportation and deployment of water in deserts can occur via passive systems that utilize sustainable resources such as the air, sun, and temperature variations. The origins of the world’s main fresh water resources are condensed from humidity in the air as rain, frost, fog, and dew as well as direct absorption from the air to the soil. Fresh water can also be produced by desalination. One estimate reported that 100 million m3 of fresh water was produced in 2015 by desalination processes at a cost of 3–5 kWh m^−3^ (Olsson 2015: 372). With a low-energy cost, desalination is an alternative for irrigation.

In a sandy dune desert, the water deposited on bare ground during nights and mornings evaporates during the day, and it is doubtful that this environment could maintain plant life (Jacobs et al. 1999; Agam and Berliner 2004, 2006; Zhuang and Zhao 2014; Zhu and Jiang 2016). The bare sandy surface kept the daytime temperature above the dew point, whereas the aboveground surfaces, with less heat capacity, cooled down below the dew point, and water was condensed on the surface (Agam and Berliner 2004). It seems plausible, but has not been reported, that the surface of a layer of fibre waste has a lower heat capacity than mineral surfaces and cools more quickly to a point below the dew point, and combined with more near surface pores than sand, it may thus absorb more water. With increasing vegetation, the plant surfaces offer larger areas for water to condense. The dew fall during nights and early mornings is important in ASAs, and even on bare sand, it was 0.1–0.2 mm night^−1^ (Jacobs et al. 1999). Non-rainfall water accounted for 13 % of the total land-surface water source in a semi-arid area (Zhang et al. 2015), and dew fall represented 19 % of the rainfall in a semi-arid coastal area (Hanisch et al. 2015). ASAs with fog conditions can condense more water from the fog (Jacobs et al. 2002). In the Qubqi Desert, a manmade crust of algae doubled the uptake of non-rainfall water (Lan et al. 2010).

### Soil organic matter

The second factor that limits agriculture is the scarcity of SOM in ASAs. In an arid desert grassland, the aboveground, belowground, and total SOM were 155.3, 95.3, and 256.3 g m^−2^, respectively. At the same site, the mean SOC density was 1.38 kg m^−2^ in the top 30 cm (Su et al. 2015). One way to increase the SOM is to add organic waste during cultivation (Zhang et al. 2009; Paustian et al. 2016). SOM is increased not only by the carbon added by the organic waste but also by vegetation sequestration of soil carbon (SSC). The estimated SSC can last up to 155 years, with a total mean SOC stock accumulation of 16.7 ± 1.5 Mg ha^−1^. SSC from the introduction of cover crops was estimated to have a potential global SOC sequestration of 0.12 ± 0.03 Pg C year^−1^, which would compensate for 8 % of the direct annual greenhouse gas emissions from agriculture (Poeplau and Don 2015). The process also works in grasslands while sustaining productive lands and reducing waste loads (DeLonge et al. 2013).

When a layer of remains from plants and trees covers the surface in ASAs, the condensed water from the humidity in the air during the night and early morning soaks into the organic material and partly remains there when the surface is dried by the sun and heat during the day (Lan et al. 2010). This moist material provides an environment for microorganisms and for organic decomposition to take place (Delgado-Baquerizo et al. 2013; Jacobson et al. 2015). The use of compost in semi-arid areas has increased TOC and the activity of beta-glucosidase linked to humic substances (Bastida et al. 2012). The process provides an environment for the cultivation of plants and trees (Reij et al. 2009).

### Cultivation techniques

The main cultivation techniques using agricultural remains have developed along two pathways. One is often referenced as the Zai method. Zai is essentially compost. Zai starts with planting in pits dug with a 50 cm diameter and a 45 cm depth. The pits are dug in the dry season and combine water harvesting and the targeted application of organic amendments (Bekunda et al. 2010; Amede et al. 2011). A pit is filled with the organic matter, and an equal amount of the material is built up as a cone around the tree trunk or base of the bush that is planted in the rainy season. This practice is iterated by preserving agricultural remains for the coming vegetation period. Zai is, in this way, a self-reinforcing process that increases SOC year by year (Poeplau and Don 2015). The agricultural remains are often mixed with animal manure (Amede et al. 2011; Moussa et al. 2016). Trees and bushes act as wind protection (Mollison 1988: 139–142). The alternative to the Zai method is to spread the material in a 0.2-m-thick layer covering the surface. Usually, the moisture from dew fall is complemented by irrigation. It is important to carefully choose the species to grow and the cultivation site. Local knowledge of species that have performed well often guides the preferred choice (Reij et al. 1998). The site is chosen according to factors such as the prevailing wind, slope, accessibility, and distance to local markets. It is often helpful to start planting trees and bushes as a wind shield for the plantation (Mollison 1988). If possible, it is preferable to also harvest saleable products from the shielding trees and bushes. The chances of successful cultivation are often enhanced by ground work to preserve water from rainfall in dams and to prevent flushing from heavy rainfall using trenches to lead the water to dams with overflow channels (Mollison 1988: 155–169).

### Carbon in textiles

Cotton is an example of textile waste. Cotton consists of approximately 90 % cellulose (Thygesen et al. 2005). Thus, an approximate estimation of the carbon released to the atmosphere when burning cellulose can be derived from the quantity of cellulose. Cellulose fibres are polymers built by molecules with the empirical formula (C_6_H_10_O_5_)_*n*_ (Chen 2014). The atomic masses are C = 12, H = 1.008, and O = 15.999. The total atomic mass of the cellulose molecule is approximately n*162 u. The six carbon atoms are 72/162 = 44.4 % of the empirical molecular weight of the cellulose. When 1 t cotton with 90 % cellulose is burned, the carbon released to the atmosphere is approximately 1000 kg * 90 % * 0.444 = 400 kg t^−1^ or 0.4 kg carbon kg^−1^ cotton. This is the same amount of carbon, 400 kg t^−1^, that can be added to the SOM using cotton as a fertilizer. The molecular weight of CO_2_ is 44, of which C is 27.27 % of the weight. Burning 1 t cotton thus releases 0.4/0.2727 = 1.47 t CO_2_ t^−1^ cotton. Synthetic polymers and wool have higher carbon contents than cotton. Thus, 400 kg t^−1^ is a lower limit when estimating the total carbon released from burning 77 100 t MSW textiles and 1550 t waste textiles, thus a low estimate from yearly textile incineration of 78 650 t is 115 600 t CO_2_ per annum.

### Carbon in ASAs

In a desert grassland, the observed aboveground biomass was1550 kgha-^1^, and the mean SOM density was 13 800 kg ha^−1^ in the top 30 cm (Su et al. 2015). These values can be used as a reference for estimates of the low and high limit values for supplementary biomass. The density of compressed textile waste is approximately 265 kg m^−3^ (Gupta at I:collect, pers. Comm.). Therefore, 17 700 kg cotton in one standard 67 m^3^ 40 ft container is enough to supplement the aboveground biomass on 11.4 ha at a cost that varies from $270 ha^−1^ on the coast of West Africa to $600 ha^−1^ in Mali. To supplement the SOM, the container covers 1.28 ha at a cost of $2400 to $5340 ha^−1^. To supplement all the SOM at one occasion is an unrealistic high ambition. In field research, the consumption of textile fibres per ha must be explored within the very wide limits described. Transportation is the main cost in the use of Swedish textile waste for soil enhancement in the Sahel area. The closer the biomass waste is to the farmland where it is used, the lower the transportation cost. Compared to incineration, the transportation cost becomes the cheapest alternative if the EU Emissions trading System (ETS) increases from $8 t^−1^ to $120 t^−1^ CO_2_. Distributed over a vegetation period of 20 years, the ETS break-even cost for the cheapest alternative is $6 t^−1^ per annum.

### Land use in ASAs

Taking into account rock fragments, soil depth, slope angle, drainage, soil texture, rainfall, aridity, protection against soil erosion, drought resistance, vegetation cover, land management, land use intensity, and policy enforcement, one analysis predicts that land prices increase when barren land is covered with vegetation, and the land use is properly distributed with secure land titles and opportunities for farmers to acquire farmland (Ferrara et al. 2012). In screening farms for sale, I compared ASA farms occupied with game or sheep and/or goat farming that were suitable for SOM enhancement and compared them with nearby farms with agriculture. The price of the cropland was 10 to 100 times higher. This comparison must be investigated further. When an ASA is developed into farmland, it can be subjected to the intervention of outside investors in the market, which calls for the protection of local interests with weak capacity to defend their legitimate interests (Cotula and Vermeulen 2009; Vermeulen and Cotula 2010; Zoomers 2010; Harvey and Pilgrim 2011). Thus, a cost–benefit analysis over time has to include the increase in land prices.

### Textile waste as a resource

The current background of the discussion of textile waste is the implementation of the Waste Framework Directive (WFD), which stipulates that a waste hierarchy shall apply as a priority order in waste prevention, management, legislation, and policy. The hierarchy is as follows: (a) prevention; (b) preparing for reuse; (c) recycling; (d) other recovery, e.g. energy recovery; and (e) disposal. Furthermore, member states should, in accordance with the waste hierarchy and for the purpose of the reduction of greenhouse gas emissions, facilitate the separate collection and proper treatment of bio-waste (European Commission 2008). In 2013, the estimated consumption of textiles for personal use in Sweden was 121 000 t; 77 100 t was left as municipal solid waste (MSW), while 28 900 t was collected by charity organizations (Östlund et al. 2015). It can be assumed that the majority of textiles in MSW is burned in power plants through incineration (Zamani et al. 2015; Östlund et al. 2015). There is no line of textile waste treatment in Sweden that could detect fractions suitable for improving SOM (Östlund et al. 2015; Zamani et al. 2015).

### Competing uses of textile waste

The recycling of textile waste is a subject for research with promising results, but there are no commercial facilities in Sweden (Östlund et al. 2015). There is also research on automatic sorting of textile waste into different fractions. One possibility is to use near-infrared reflectance spectroscopy (NIR) to identify fractions of the waste. One demonstration facility, Fibersort, is scheduled to start in 2017, which shall have a yearly capacity of 5000 t (Beton et al. 2014; Östlund et al. 2015). Using hyperspectral imaging in a broader spectrum before using NIR is even more promising, but is not yet available (Humpston et al. 2014). Automatic sorting by the use of NIR provides the opportunity to estimate the carbon content of waste fractions (Thuriès et al. 2005; Peltre et al. 2011).

There is a hierarchical sequence for used textiles depending on the deterioration of the fibre quality by rounds of recycling. The proposed best practice in Sweden is reuse followed by the recycling of cotton fibres > lyocell fibres > viscose fibres > incineration (Östlund et al. 2015). The suggestion in this study is to expand the last transition by one step from viscose fibres > incineration to two steps of viscose fibres > SOM enhancement > incineration. At present, the alternative, after the recycling options are exhausted, is incineration. Thus, the evaluation of the suggestions in this study is reduced to comparing SOM enhancement to incineration.

Charity organizations leave 1550 t for incineration. These textiles have toxic fractions, mainly from colours, and of a mix of natural fibres and synthetic fibres of fossil origin (Östlund et al. 2015). The majority of fibres with fossil origin resist biological decomposition. These fractions are not suitable for SOM enhancement. The quantity of the remaining fraction of pure cellulose fibres is difficult to estimate, but it seems plausible that starting with 1550 t of textiles from charity would enable field research. The standard practice for this fraction is incineration, where the recovered energy is distributed in district heating networks and in generating electrical power. The energy recovered is approximately 17 000 MJ t^−1^, and the efficiency of Swedish power plants averages 97 %. The conversion factor of 1 MJ = 0.278 kWh, and an assumption for this approximation that all energy from the plants is delivered as electrical power provides 17 000 MJ t^−1^ * 97 % * 0.278 = approximately 4.6 MWh t^−1^ cotton. With an average price of $ 0.04 kWh^−1^, electrical power gives an income of approximately $166 t^−1^ of textiles. I have no figure on the production costs of incineration. One estimate is that the overall result at a plant was 33 %, which would give a revenue of $55 t^−1^. ETS has a price on the secondary market of approximately $10 t^−1^ CO_2_. Thus, the ETS cost is approximately $15 t^−1^ textile waste, which is approximately 38 % of the $40 t^−1^ charged to customers for burning textiles at incineration plants. The lost revenue for not burning the textiles can be estimated as $80 t^−1^ or $ 0.08 kg^−1^.

With the comparison of textiles entering an incineration facility, there is already a transportation cost and a fee for incineration of $70 t^−1^. Let this transportation cost + the incineration fee equal the transportation to the Swedish harbour Göteborg. Transportation of one 40 ft container from Göteborg to West Africa costs approximately $3000 and to sub-Saharan Bamako in Mali approximately $6900. One 40 ft container can hold 17 700 kg biomass of compressed cotton fibres with an organic carbon content of 7080 kg. Thus, this transportation costs vary between $170 and $390 t^−1^ cotton fibres. The combination of the lost revenue from incineration and the cost for transporting textile waste to West Africa or a sub-Saharan inland destination is estimated as $250 to $470 t^−1^ cotton fibres. The labour cost to distribute the textiles from the container to the soil, irrigation, and other processes is not estimated as it is a part of the normal agricultural work, e.g. by Zai farming. The burning of 17 700 kg cotton releases 26 ton CO_2_ into the air at an ETS cost of $10 t^−1^. In an ETS interval of $250–$470 ton^−1^ CO_2_, there is a break-even point to the lost revenue from incineration and the transportation cost to West Africa and Mali. Calculated over a 20-year vegetation period, the break-even ETS costs is in the interval $13-24. A British study of the market value of textile fibres for soil re-enforcement reported $570 t^−1^ (Humpston et al. 2014). If the textile fibres are bought at this price, then the price at a West African harbour is $820 t^−1^. However, there is no market reported for textiles as soil reinforcement in Sweden (Östlund et al. 2015).

## Discussion

Studies report that water absorption from dew and air humidity increases with an increase in the organic surface layer (Fischer et al. 2012). Thus, it is interesting to investigate possibilities to increase the surface organic layer by adding organic matter to increase water absorption in ASAs. The second reason for such SOM enhancement is that large ASAs have no or a very low amount of SOM in the surface layers. Humid SOM offers microbial activity that promotes plant life (Delgado-Baquerizo et al. 2013; Jacobson et al. 2015). In ASAs, the remains of local agricultural have been used to enhance SOM for tree planting and agriculture (Reij and Pearce 2008; Reij et al. 2009). This activity suggests that fibre waste could be used for these purposes. There are three main environmental mitigation benefits of such agriculture. The first benefit is the conservation of carbon in the textiles compared to the current burning of the waste (Östlund et al. 2015; Zamani et al. 2015). The second is the sequestration of soil carbon (SSC) from atmospheric CO_2_ by plant life in previously barren land (Zhang et al. 2009; Poeplau and Don 2015; Paustian et al. 2016). Because the SOM layer is kept on the ground for years, the duration of SSC is estimated to be 155 years (Poeplau and Don 2015). The third benefit is increased water absorption (Jacobs et al. 1999; Agam and Berliner 2004, 2006; Fischer et al. 2012; Zhu and Jiang 2016). Aside from daily water from humidity in the air, irrigation is needed for cultivation (Jacobs et al. 1999; Agam and Berliner 2004, 2006; Zhu and Jiang 2016). The water sources for irrigation can be enhanced by earthworks for water conservation and storage (Mollison 1988: 155–170; Reij 1988; Ostberg and Reij 1998; Reij et al. 2005).

According to the WFD hierarchy, the quantity of burned textile waste in Sweden calls for practices to prepare for reuse. Following the WFD, there is current work on how to treat waste, where the last steps in a recovering process are burning and deposition (European Commission 2008; Beton et al. 2014; Östlund et al. 2015). The WFD requires that member states shall establish processes to treat each flow of waste so that the waste can be reused, recycled, or processed for energy conservation. Energy conservation in Sweden is mainly performed by incineration in power plants (Östlund et al. 2015; Zamani et al. 2015). The suggestion in this study is to introduce a step of SOM enhancement before incineration to be applied on the fraction of waste where the fibres are so worn that they are not suitable for recycling. There are costs to collect and sort textile waste, but these costs have to be covered anyway due to WDF. There are promising results of developing automatic sorting for textile fibres, which could decrease the costs of sorting the worn out fibres (Beton et al. 2014; Östlund et al. 2015). Thus, when investigating the competing uses of the worn out fibre fraction, the competition in Sweden is incineration, with its use for energy recovery. As shown here, the cost for SOM enhancement by textile fibres from Sweden is dominated by transportation costs, and a break-even ETS of $250–$470 t^−1^ pays for the delivery of worn out fibres from Sweden to West Africa and Mali as an example of an inland sub-Saharan destination. Cultivation over 20 years distributes the break-even ETS cost to $13–24. This cost has to be taken into account when planning for field research. Thus, it is important to choose an area with low transportation costs.

Placing fibre waste as a layer on arid surfaces requires the means of acquiring, transporting, and spreading it appropriately. There are at least two main options for mobilizing these resources: one is using volunteers and international aid organizations, and the other is leaving it to market forces. The reasons for using volunteers and aid organizations are that the collection and distribution of textile waste offer opportunities to individuals and aid organizations to contribute to the solution to an environmental CO_2_ problem. The support for environmental improvements is high in the Swedish population. The collection of fibres such as textiles offers an alternative to mundane apathy when faced with global warming and ocean acidification as consequences of the increasing CO_2_ density in the atmosphere. As an example, there are two large multi-national textile companies in Sweden that offer their customers the option of donating used textiles for recycling of the fibres. A fraction of these textiles is considered to be to worn out and thus incinerated. There are already charity organizations collecting textiles, and others secure access of fresh water for populations in need. Thus, the mobilization towards cultivation in ASAs seems plausible.

Projects to introduce farming practices such as Zai have had support, mostly for education, by NGOs, and it has also been promoted by engaging a vast variety people with local knowledge and experience (Reij 1988, 1998; Ostberg and Reij 1998; Reij et al. 1998; Sendzimir et al. 2011). The NGO Commonland is working to build a suitable mobile training camp that can be deployed where needed (Liu, pers. comm.). There is no reason that the large engagement in the development of agriculture in the Sahel should vanish in the coming years.

The reasons for using market forces to bear the costs are possible profits. There are two main profit sources. One source is the possible sale of products from agriculture and forestry, and the other is the estimated tenfold increase in land prices from the transformation of almost barren desert to agricultural land (Cotula and Vermeulen 2009; Vermeulen and Cotula 2010; Zoomers 2010; Harvey and Pilgrim 2011; Ferrara et al. 2012).

## Conclusion

The reports on possible environmental mitigation by preventing the release of CO_2_ from burning textiles and the sequestration of carbon in soil organic matter give reason to start field research on the use of textiles as a soil enhancement. There is a time window to exploit this possibility as there are requirements by the European Commission to establish routines for waste recycling and reuse. Simultaneously, there are developing techniques for the automatic sorting of textiles into fractions suitable for different reuse and recycling possibilities. One such possibility is to sort out the fraction suitable for soil enhancement. The time window may be closed by the development of other strategies due to the shortage of knowledge of possible soil enhancements. Thus, I suggest that field research is initiated as soon as possible.
